# Cognitive training in neurological disorders: meta-analytic evidence for behavioral gains and neural correlates

**DOI:** 10.1186/s12993-026-00323-3

**Published:** 2026-03-02

**Authors:** Geng Li, Yang Liu, Chengzhen Liu, Yihan Zhang

**Affiliations:** 1 School of Psychology, Research Center for Exercise and Brain Science, Shanghai University of Sport, Shanghai, China; 2 School of Physical Education, Hunan Normal University, Changsha, China; 3 School of Sports Science, Jishou University, Jishou Jishou, China; 4 Faculty of Psychology, Southwest University, Chongqing, China

**Keywords:** Cognitive training, Neuroimaging, Neuroplasticity, Meta-analysis, Neurological disorders

## Abstract

**Background:**

Cognitive decline in neurological disorders substantially impairs daily functioning and quality of life, underscoring the need for effective non-pharmacological interventions. We aimed to quantify the behavioral benefits of cognitive training, characterize convergent patterns of task-related brain activation changes, and examine moderators of the neural responses underlying training effects.

**Methods:**

We conducted a meta-analysis of 21 task-based neuroimaging studies. Behavioral outcomes were synthesized using multivariate meta-analysis, while neural changes were examined with seed-based d mapping (SDM) to identify spatially consistent activation differences between training and control groups. Moderator analyses evaluated training parameters, study designs, and participant characteristics, and brain–behavior associations were assessed to link regional activation changes with cognitive improvements.

**Results:**

Cognitive training produced a significant, moderate improvement in cognitive task performance (Hedges’ g = 0.451, 95% CI 0.207–0.696; *p* < .001). Neuroimaging meta-analysis showed increased task-evoked activation after training in the bilateral precuneus and the left precentral gyrus (L. PreCG) relative to controls. Only precuneus activation increases were associated with behavioral gains. Moderator analyses indicated reduced precuneus activation but increased L. PreCG activation during transfer relative to trained tasks, and reduced L. PreCG activation for adaptive versus fixed-difficulty training. Passive controls also showed stronger L. PreCG activation changes than active controls, whereas participant characteristics and training dose showed no significant effects.

**Conclusions:**

Cognitive training improved cognitive task performance and was accompanied by a convergent activation signature centered on the bilateral precuneus and the L. PreCG in neurological disorders. Precuneus engagement tracked cognitive gains, highlighting it as a candidate neural marker of training-related plasticity in this population.

**Supplementary Information:**

The online version contains supplementary material available at 10.1186/s12993-026-00323-3.

## Introduction

Neurological disorders encompass diverse conditions, including neurodegenerative diseases, cerebrovascular disease, traumatic brain injury, and demyelinating disorders [1]. Despite distinct underlying pathology, these conditions frequently converge on disruptions of large-scale cognitive networks and are commonly accompanied by progressive decline in attention, executive function, and memory that substantially compromises daily functioning and may ultimately be fatal [2]. Dementia, the most prevalent clinical manifestation of such decline, currently affects 57.4 million people worldwide and is projected to reach 153 million by 2050 [3]. Beyond their profound impact on quality of life, neurological disorders impose major societal and economic costs, with dementia-related unpaid caregiving in the United States alone valued at over 339.5 billion annually [4]. Pharmacological options remain limited, with few approved agents and modest efficacy, while emerging anti-amyloid therapies have shown only partial benefit [5, 6]. Accordingly, there is growing interest in non-pharmacological interventions as scalable strategies to preserve cognitive function. Overall, cognitive decline across neurological disorders is often accompanied by dysfunction in distributed control and associative networks, providing a systems-level rationale for subsequent investigation [7, 8].

Among non-pharmacological approaches, cognitive training (also referred to as cognitive remediation) is a behavioral intervention designed to improve cognitive abilities through repeated engagement in structured tasks [9]. In recent years, cognitive training has attracted considerable attention in research on cognitive impairment and decline [10]. This interest stems from accumulating evidence that cognitive training can induce structural and functional plasticity in the brain [10–13], and promote behavioral improvements by modulating task-related neural activation [14, 15]. Nevertheless, findings remain heterogeneous. For example, one study reported increased frontal activation following cognitive training alongside cognitive improvements [16], whereas another study observed behavioral gains accompanied by increased activation in the left anterior temporal lobe [17]. Such inconsistencies may stem from differences in sample size, training parameters, and experimental design across studies.

Given divergent findings across individual studies, rigorous synthesis is needed [18]. Meta-analysis enables integration across studies to identify consistent effects and delineate convergent neural correlates of training, and facilitates the development of functional brain models by elucidating underlying mechanisms [19]. Training-related plasticity in task-related activation may manifest as either enhancement, reflecting increased recruitment of task-relevant regions, or efficiency, characterized by reduced activation alongside improved performance [17]. For example, a meta-analysis of cognitive training in healthy individuals reported efficiency-related reductions in task-related activation [12]. By contrast, the Compensation-Related Utilization of Neural Circuits Hypothesis (CRUNCH) posits that brain damage may prompt compensatory recruitment of additional regions to offset reduced efficiency in primary task networks [20]. Given constrained neural resources and impaired network efficiency in neurological disorders, we therefore expected training-related neural changes to manifest predominantly as enhanced recruitment, reflected by increased task-related activation. At the same time, because learning can also reduce processing demands, we additionally tested whether training-related efficiency patterns could be observed. To date, however, no study has systematically examined whether cognitive training yields a convergent neural signature in neurological disorders, or whether observed changes more closely align with enhanced recruitment, greater efficiency, or a combination of both.

Accordingly, this study quantified cognitive training–related changes in cognitive task performance and characterized convergent patterns of task-related activation in neurological disorders. We then tested whether neural responses varied with participant characteristics, training parameters, and study design features. Finally, we related regional activation changes to behavioral improvement to identify candidate neural correlates that most consistently track training gains.

## Methods

In line with best-practice recommendations for neuroimaging meta-analyses [21], we systematically examined task-related brain activation changes associated with cognitive training in individuals with neurological disorders. Literature search and study selection followed the PRISMA framework, with full methodological details available in Supplementary Table S1. The protocol was prospectively registered in PROSPERO (CRD42024599205).

### Information sources, search strategy, and study selection process

We searched five electronic databases (PubMed, Web of Science, PsycINFO, MEDLINE, and Embase), with the final update completed on 12 November 2024, using a comprehensive strategy targeting neuroimaging and cognitive training (see Supplementary Table S2). Reference lists of all eligible articles were also manually screened to capture additional studies. After removing duplicates in EndNote (version 21), titles and abstracts were assessed against predefined eligibility criteria, followed by full-text review for final inclusion. To ensure methodological rigor, two authors (YL and CL) independently conducted all screening steps, with disagreements resolved through discussion or, when necessary, consultation with a senior author (GL).

### Eligibility criteria

Studies were eligible for inclusion if they met the following criteria: (1) reported stereotactic coordinates of task-related brain activation changes induced by cognitive training; (2) employed whole-brain analyses; (3) used either within-subject (pre–post) or between-subject designs, with control conditions including active comparators (e.g., mental leisure activities, educational programs, low-challenge sham training) or passive comparators (e.g., waitlist); and (4) involved participants with neurological disorders such as neurodegenerative diseases, cerebrovascular disease, traumatic brain injury, or demyelinating conditions [22, 23].

### Data extraction and summary of outcomes

Two authors (YL and CL) independently screened all records for eligibility and extracted data using standardized forms. Extracted variables included participant characteristics, details of cognitive training protocols, control conditions, and study design features. For SDM analyses, peak activation coordinates and corresponding statistical indices (e.g., t values, z scores, p values) were collected. In studies reporting multiple tasks, populations, or training groups, peak coordinates were extracted separately for each condition to ensure precision and avoid conflation [24].

## Meta-analysis

### Multivariate meta-analysis

The multivariate meta-analysis of behavioral outcomes was conducted in RStudio (version 1.4.1106) using the *metafor* and *clubSandwich* packages. Unlike univariate approaches, multivariate meta-analysis incorporates multiple effect sizes from the same study, accounts for intercorrelations among outcomes, and provides more precise estimates [25]. A three-level hierarchical structure was adopted, with individual outcomes (Level 1) nested within samples (Level 2), and samples nested within studies (Level 3). Random effects were specified at all levels to model nested dependencies, and the within-study correlation among cognitive outcomes was fixed at 0.5 according to guidance from the Cochrane Handbook for Systematic Reviews of Interventions [26]. The robustness of the multivariate meta-analytic findings was further evaluated by re-estimating the models under alternative assumptions of within-study correlations (*r* = .3, 0.7, 1) [27].

Effect sizes were calculated as standardized mean differences (SMDs) using Hedges’ g, which corrects for small-sample bias [28]. For each outcome, Hedges’ g was derived from the between-group difference in pre-to-post change scores. When change-score standard deviations were not reported, they were computed from pre- and post-intervention standard deviations by imputing a pre–post correlation of *r* = .5, following established guidance [29]. For outcomes such as reaction time or error rates, values were reverse-coded where necessary so that positive effect sizes consistently reflected cognitive improvement [30]. Following established practice [31], effect sizes were aggregated across both global cognition and specific cognitive subdomains to evaluate the overall impact of cognitive training. Effect sizes were interpreted according to conventional thresholds as small (≤ 0.2), medium (0.2–0.8), or large (≥ 0.8) [32].

### SDM meta-analysis

This study employed Seed-based d Mapping (SDM, version 5.15) to examine task-related activation changes induced by cognitive training [33]. Following established SDM guidelines and prior applications in meta-analyses [34, 35], additional procedures were adopted when statistical values or thresholds were incomplete. Specifically, when only coordinates were reported, thresholds were inferred from estimated effect sizes; conversely, when only thresholds were available, effect sizes were derived from those data. Reported Z or P values were converted to t statistics using the SDM online converter, with stereotactic space and statistical metrics automatically standardized by the software.

Image preprocessing followed SDM recommendations, using a 20-mm full-width at half-maximum (FWHM) isotropic Gaussian kernel and 500 randomizations. For studies reporting multiple contrasts, the “combine image” function was applied to generate composite maps reflecting the mean effect size and variance across contrasts [36, 37]. Statistical significance was defined at an uncorrected threshold of *p* < .0001, which has been shown to approximate corrected thresholds in SDM analyses [24]. Clusters were considered significant if they exhibited a peak SDM-Z ≥ 1 and comprised ≥ 10 contiguous voxels. The robustness of the SDM meta-analytic results was evaluated using jackknife sensitivity analyses. Potential small-study effects were further assessed for each significant cluster using Egger’s regression test.

### Moderator analyses

To examine categorical moderators of training effects, we conducted comparative analyses using binary classifications. Specifically, we tested training domain (multi- vs. single-domain training), training paradigm (process-based vs. strategy-based training), delivery mode (group-based vs. individual-based training), difficulty regulation (adaptive vs. non-adaptive difficulty training), design type (between-subject vs. within-subject design), control condition (active vs. passive controls), and task type (trained vs. transfer tasks). In addition, regression analyses were performed to assess continuous moderators, including mean participant age (years), sex distribution (% female), session duration (minutes), program length (weeks), total training dose (minutes), training frequency (sessions/week), and adherence (%). To mitigate false-positive findings arising from multiple testing, moderator inferences were restricted to clusters that were already significant in the overall SDM analysis, and results were reported only when the same clusters also demonstrated significant moderation effects. The same stringent SDM threshold (*p* < .0001) was applied throughout to limit false positives, consistent with prior work [38].

### Brain–behavior associations

This study further examined the relationship between training-induced changes in task-related brain activation and cognitive performance gains across studies. Brain–behavior associations were tested by linking effect sizes of behavioral improvement (Hedges’ g) with activation changes in clusters identified in the overall analysis. Associations were considered significant at *p* < .05, a threshold chosen to balance sensitivity and specificity given the exploratory nature of brain–behavior analyses in meta-analysis [21, 34].

### Quality assessment

To enhance the methodological rigor of this systematic review, we assessed the reporting quality of included neuroimaging studies using a 17-item checklist adapted from prior work [39]. The checklist encompassed key domains such as participant characteristics, study design, data acquisition, preprocessing, statistical analysis, and reporting of conclusions. Detailed criteria are presented in Supplementary Table S3, and the results of the quality assessment are summarized in Supplementary Table S4.

## Results

### Study selection

**Fig. 1:**
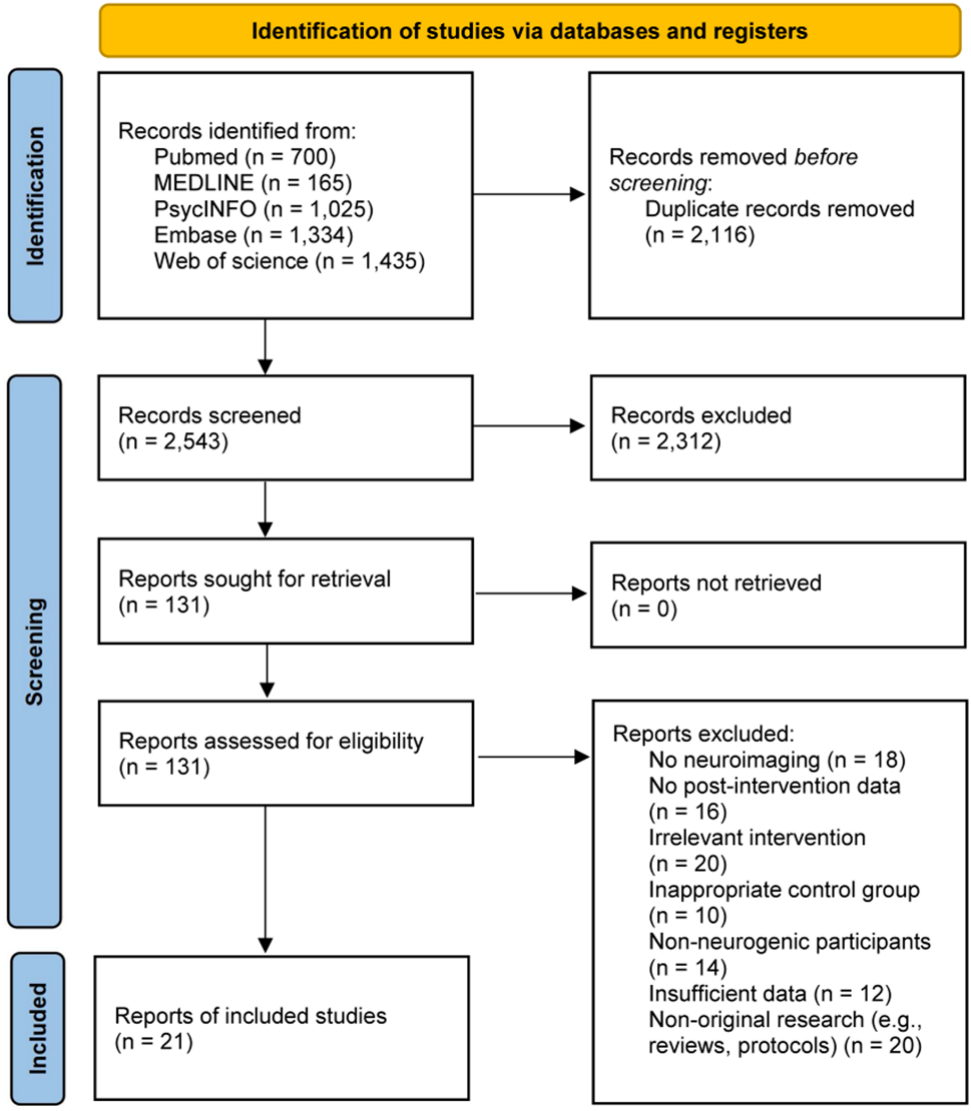
PRISMA flowchart detailing the process for screening and including relevant studies

A systematic search was conducted across PubMed, Web of Science, APA PsycINFO, MEDLINE, and Embase using predefined keywords related to neuroimaging and cognitive training. The search initially identified 4,659 records. After the removal of 2,116 duplicates, 2,543 unique records remained. Title and abstract screening excluded 2,312 articles, resulting in 131 full-text articles assessed for eligibility. Ultimately, 21 studies met the inclusion criteria and were incorporated into the final meta-analysis (Fig. 1).

### Study characteristics

Across the 21 studies included in this meta-analysis [17, 40–59] a total of 502 participants (mean age = 56.95 years; 52.72% female) were analyzed. On average, cognitive training programs comprised 15.08 sessions with a total mean duration of 834.31 min, delivered at a frequency of 2.82 sessions per week, with an adherence rate of 92.3%. Each session lasted approximately 53.18 min. Detailed study characteristics are provided in Supplementary Table S4.

### Meta-analysis

#### Overall analysis

**Fig. 2:**
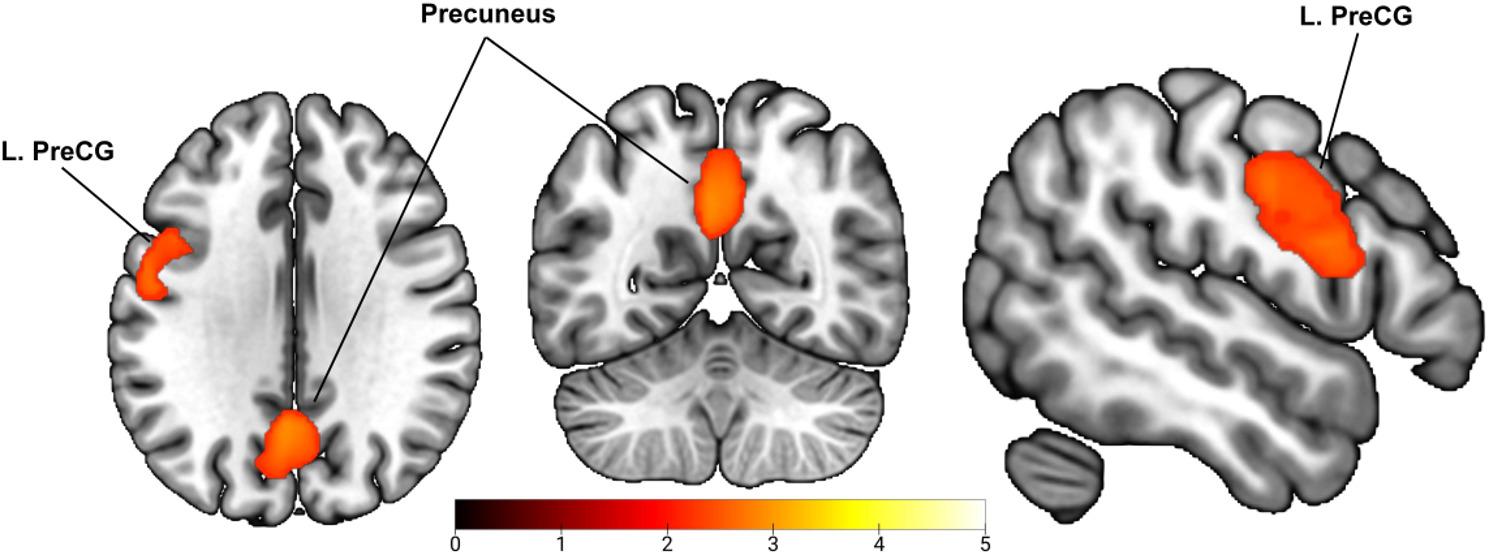
SDM meta-analysis results of task-related brain activation changes induced by cognitive training. Brain regions identified by the SDM analysis were thresholded at uncorrected *p* < .0001, which is considered to provide a level of stringency comparable to corrected thresholds [24]. Clusters were considered significant if they exhibited a peak SDM z ≥ 1 and comprised ≥ 10 contiguous voxels. Red regions indicate increased activation. L. PreCG = left precentral gyrus

A multivariate meta-analysis of 13 studies revealed that cognitive training produced significant improvements in cognitive task performance relative to controls, with a moderate pooled effect size (Hedges’ g = 0.451, 95% CI 0.207–0.696; t = 3.745; *p* < .001, I^2^ = 35.955%). Complementarily, an overall SDM meta-analysis of 21 studies identified two significant clusters of training-related changes in task-evoked brain activation relative to controls (Table 1; Fig. 2), including the bilateral precuneus, showing a significant increase in activation (SDM-Z = 2.932, voxels = 1,112, *p* < .0001; I² = 7.445%), and the left precentral gyrus (L. PreCG), which exhibited a similar increase (SDM-Z = 2.753, voxels = 707, *p* < .0001; I² = 1.821%).

**Table 1:** Brain regions with significant differences in task-related activation between cognitive training and controls in the overall analysis and comparative analysis

MNI coordinate	SDM-Z	*p*	Voxels	Description
Overall analysis				
Cognitive training > controls				
-4, -60, 34	2.932	~ 0	1112	Bilateral precuneus
-52, 6, 20	2.753	~ 0	707	Left precentral gyrus
Comparisons analysis				
Adaptive difficulty < fixed difficulty				
-54, 4, 34	–2.084	0.000025809	104	Left precentral gyrus
Transfer task > Trained task				
-54, -4, 32	1.604	0.000005186	63	Left precentral gyrus
Transfer task < Trained task				
6, -58, 38	–2.517	~ 0	804	Bilateral precuneus
Passive compare > Active compare				
-52,4,20	1.608	~ 0	159	Left precentral gyrus

#### Moderator analyses

The comparative analyses revealed no significant differences in task-related brain activation associated with training domain, training paradigm, delivery mode, or design type. By contrast, several moderators exerted significant effects. Fixed-difficulty training, compared with adaptive difficulty, was associated with greater activation in the L. PreCG (SDM-Z = 2.084; 104 voxels). Passive controls similarly elicited stronger activation than active controls in the L. PreCG (SDM-Z = 1.608; 159 voxels). Task type also modulated training effects: transfer tasks, relative to trained tasks, recruited the L. PreCG (SDM-Z = 1.604; 63 voxels), whereas trained tasks preferentially engaged the bilateral precuneus (SDM-Z = 2.517; 804 voxels).

Regression analyses further indicated that training-induced changes in task-related activation were not significantly moderated by continuous variables, including participant age, session duration, program length, total program duration, training frequency, sex, or training adherence.

#### Brain–behavior associations

**Fig. 3:**
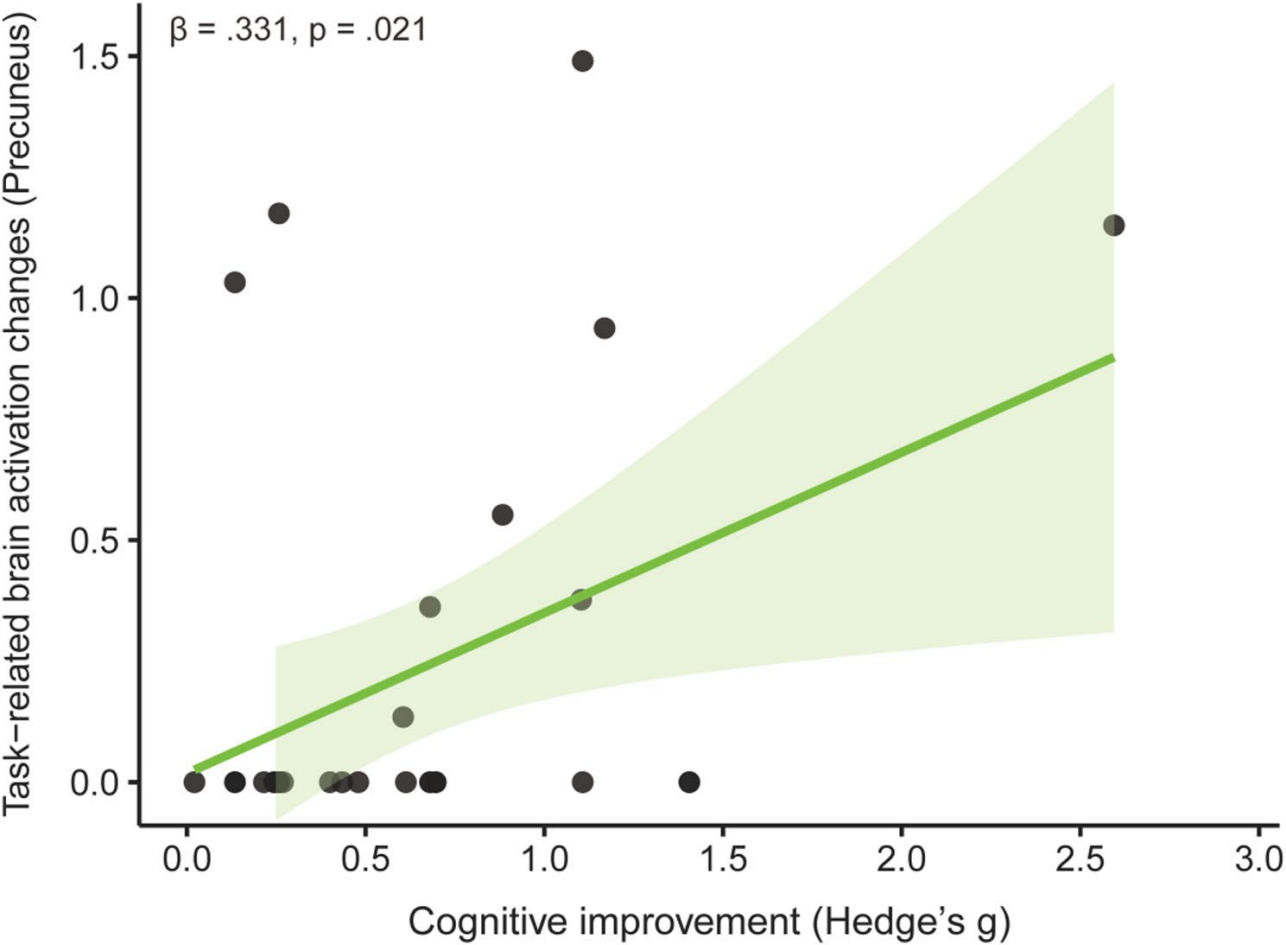
Regression results showing associations between cognitive improvement and training-induced activation changes in the bilateral precuneus. Each dot represents an effect size, with larger dots indicating larger sample sizes. The regression line is shown in green, with the shaded area denoting the confidence interval. The meta-regression SDM value reflects the proportion of studies reporting brain activation changes near a given voxel; therefore, it is expected that some study values are at 0 or close to + 1, rather than aligning closely with the regression line [38]

Brain–behavior relationships were examined using regression analysis. Improvements in cognitive task performance were significantly associated with training-induced changes in task-related activation within the bilateral precuneus (β = 0.331, *p* = .021; Fig. 3), whereas no significant associations emerged for the L. PreCG (*p* > .05).

#### Reliability analysis results

As shown in Supplementary Table S5, jackknife sensitivity analyses indicated high replicability of cognitive training–related task-evoked activation changes, with the main findings remaining consistent across all leave-one-out study combinations. Consistent with this pattern, Egger’s tests indicated no evidence of funnel plot asymmetry for the bilateral precuneus (β = 0.28, t = 0.46, *p* = .65) or the L. PreCG (β = 0.56, t = 0.84, *p* = .405), as shown in Supplementary Figs. 1–2. Likewise, Supplementary Table S6 demonstrated the robustness of cognitive training–related improvements in cognitive performance, with pooled effects remaining stable across all assumed within-study correlation values.

## Discussion

This meta-analysis of 21 neuroimaging studies in individuals with neurological disorders demonstrates that cognitive training not only improved cognitive task performance but also induced task-related brain activation increases in the bilateral precuneus and the L. PreCG. Among these regions, only bilateral precuneus activation was associated with cognitive improvement. Moderator analyses further revealed that training design modulated neural responses. Fixed-difficulty protocols and active control conditions preferentially recruited the L. PreCG. In contrast, transfer tasks elicited reduced activation in the bilateral precuneus compared with trained tasks. No moderating effects emerged for participant characteristics or training parameters. These findings delineate the neural architecture of cognitive training-induced plasticity in neurological disorders.

This study demonstrates that cognitive training not only produces moderate improvements in cognitive task performance but is also accompanied by increased activation in the bilateral precuneus and the L. PreCG. While the behavioral gains are broadly consistent with previous work [60], the present findings add convergent evidence regarding their neural correlates. The precuneus, a central associative hub linking parietal and frontal regions, is a critical node within the frontoparietal network and functions as an efficient “small-world” connector that supports large-scale information integration [61]. As a convergence zone between the default mode and task-positive networks, its increased activation may reflect training-related enhancements in attentional allocation and integrative processing [62, 63]. From the perspective of the scaffolding theory of aging and cognition (STAC), individuals with neurological disorders may engage alternative neural circuits, particularly within the default mode network (DMN), to sustain cognitive performance [64]. In contrast, increased activation in the L. PreCG is more likely to reflect motor preparation and response output related to button-press demands rather than training-specific cognitive processing [65, 66], consistent with the fact that L. PreCG changes did not covary with behavioral gains. Ultimately, training-related activation changes showed a differentiated pattern, with bilateral precuneus upregulation co-occurring with cognitive improvement, whereas increased L. PreCG activation more likely reflected an accompanying adjustment to response-related demands.

This study identified several factors that significantly moderated training-related neural changes. Specifically, adaptive training, relative to fixed-difficulty training, was associated with weaker activation in the L. PreCG, consistent with reduced reliance on premotor resources and the neural efficiency hypothesis, whereby skill acquisition following repeated practice can be achieved with fewer neural resources [67]. Similarly, trained tasks showed reduced recruitment of the L. PreCG, whereas transfer tasks were characterized by increased L. PreCG recruitment together with weaker activation in the bilateral precuneus. Given that bilateral precuneus engagement tracked behavioral improvement, diminished precuneus recruitment during transfer more likely reflects weaker engagement of a gain-relevant associative hub rather than successful efficiency. Accordingly, this pattern may help account for the limited generalization of training benefits beyond the trained task, extending prior behavioral evidence [60]. Finally, passive control conditions exhibited stronger L. PreCG activation than active controls. While this difference may partly reflect expectancy-related effects [68], it may also arise because active control tasks typically involve cognitive engagement and motor responding, which could reduce premotor recruitment via incidental practice. Together, these findings suggest that differences between passive and active controls may reflect not only expectancy effects but also variation in the cognitive and motor demands embedded in the control condition.

In contrast, several potential moderators, including participant characteristics and training dosage, did not show reliable effects, which warrants further consideration. The absence of age-related moderation may be consistent with a non-linear, potentially inverted U-shaped relationship between age and training outcomes, as suggested by prior work [30, 69, 70]. Moreover, because all included samples comprised individuals with neurological disorders, any age-related variation may be difficult to disentangle from disease-related neural changes, potentially obscuring simple linear associations. Likewise, the lack of a dosage effect suggests that greater training exposure does not necessarily translate into proportionally larger benefits. One possibility is that training responses may plateau beyond a certain intensity, yielding diminishing returns with additional practice [71]. Such ceiling-like patterns could be particularly relevant in neurological populations, where structural pathology may limit the range of functional adaptation [72–74]. Together, these observations highlight the importance of considering biological constraints and non-linear trajectories when designing cognitive training interventions for clinical populations.

This study has several limitations that should be considered in future research. First, due to the limited number of eligible studies, we did not implement formal multiple-comparisons correction; although we applied a stringent uncorrected threshold in line with common practice in the field [34, 35] to reduce false positives, some significant findings may still be susceptible to inflated Type I error. Future studies should include larger samples and adopt more systematic multiplicity control to improve the robustness and reproducibility of the conclusions. Second, to avoid sparse subgroups and ensure stable estimation, several moderators were dichotomized for comparative analyses, which may have masked finer-grained distinctions and limited interpretability. Future studies with larger samples and individual-level data will allow more granular coding and yield more robust effect estimates. Finally, inconsistent reporting of key clinical and imaging variables limited confounder control across studies. Standardized reporting and targeted sensitivity analyses in future datasets will be essential to strengthen robustness and generalizability.

## Conclusions

By integrating 21 task-based neuroimaging studies in individuals with neurological disorders, this meta-analysis demonstrates that cognitive training yields moderate improvements in cognitive task performance and induces convergent task-related activation changes in the bilateral precuneus and the L PreCG. Notably, only the increase in bilateral precuneus activation was associated with cognitive gains, highlighting the precuneus as a robust neural correlate of training-related plasticity. Moderator analyses further indicated that study design features, particularly difficulty regulation, task type, and control condition, were associated with variability in neural responses, whereas participant characteristics and training dose showed no reliable effects. Together, this study delineates a consistent neural activation signature of cognitive training in neurological disorders and shows that increased precuneus activation co-occurs with behavioral gains, providing convergent support for the precuneus as a candidate neural marker of training-related plasticity.

## Supplementary Information

Below is the link to the electronic supplementary material.

Supplementary Material 1.

## Data Availability

All data supporting the findings of this study are available from the corresponding author upon request.

## Abbreviations

DMN: Default mode network
L. PreCG: Left precentral gyrus
PRISMA: Preferred reporting items for systematic reviews and meta—analyses
SDM: Seed—based d mapping
SMDs: Standardized mean differences
STAC: Scaffolding theory of aging and cognition
CRUNCH: Compensation—related utilization of neural circuits hypothesis
FWHM: Full width at half maximum
