# Supplementary material for: Cognitive training in neurological disorders: meta-analytic evidence for behavioral gains and neural correlates
Source: Behav Brain Funct. 2026 Mar 2;22:25. doi: 10.1186/s12993-026-00323-3 (PMC13248290; doi:10.1186/s12993-026-00323-3)
Supplement: Supplementary file 1 — Supplementary Material 1. [file 12993_2026_323_MOESM1_ESM.pdf]

Supplementary Materials

Contents

Supplementary Table S1. PRISMA 2020 Checklis 1

Supplementary Table S2. Electronic database search 5

Supplementary Table S3. Quality assessment checklist 8

Supplementary Table S4. Participant, intervention, result and quality of included studies 10

Supplementary Table S5. Results of the jackknife analysis in all included studies........ 18

Supplementary Table S6. Sensitivity results of multivariate meta-analysis across assumed within-study correlations........ 19

Supplementary Figure S1. Sensitivity results of multivariate meta-analysis across assumed within-study correlations........ 19

Supplementary Figure S2. Sensitivity results of multivariate meta-analysis across assumed within-study correlations........ 19

Table S1

PRISMA 2020 Checklis

Section and Topic	Item #	Checklist item	Location where item is reported
**TITLE**			
Title	1	Identify the report as a systematic review.	Manuscript Page 1
**ABSTRACT**			
Abstract	2	See the PRISMA 2020 for Abstracts checklist.	Manuscript Page 1
**INTRODUCTION**			
Rationale	3	Describe the rationale for the review in the context of existing knowledge.	Manuscript Pages 2–3
Objectives	4	Provide an explicit statement of the objective(s) or question(s) the review addresses.	Manuscript Page 3
**METHODS**			
Eligibility criteria	5	Specify the inclusion and exclusion criteria for the review and how studies were grouped for the syntheses.	Manuscript Pages 3–4
Information sources	6	Specify all databases, registers, websites, organisations, reference lists and other sources searched or consulted to identify studies. Specify the date when each source was last searched or consulted.	Manuscript Page 4
Search strategy	7	Present the full search strategies for all databases, registers and websites, including any filters and limits used.	Manuscript Supplementary Materials Pages 5–9
Selection process	8	Specify the methods used to decide whether a study met the inclusion criteria of the review, including how many reviewers screened each record and each report retrieved, whether they worked independently, and if applicable, details of automation tools used in the process.	Manuscript Pages 3–4
Data collection process	9	Specify the methods used to collect data from reports, including how many reviewers collected data from each report, whether they worked independently, any processes for obtaining or confirming data from study investigators, and if applicable, details of automation tools used in the process.	Manuscript Page 4

Data items	10a	List and define all outcomes for which data were sought. Specify whether all results that were compatible with each outcome domain in each study were sought (e.g. for all measures, time points, analyses), and if not, the methods used to decide which results to collect.	Manuscript Pages 4–5
	10b	List and define all other variables for which data were sought (e.g. participant and intervention characteristics, funding sources). Describe any assumptions made about any missing or unclear information.	Manuscript Pages 4–5
Study risk of bias assessment	11	Specify the methods used to assess risk of bias in the included studies, including details of the tool(s) used, how many reviewers assessed each study and whether they worked independently, and if applicable, details of automation tools used in the process.	Manuscript Pages 6–7
Effect measures	12	Specify for each outcome the effect measure(s) (e.g. risk ratio, mean difference) used in the synthesis or presentation of results.	Manuscript Pages 5–6
Synthesis methods	13a	Describe the processes used to decide which studies were eligible for each synthesis (e.g. tabulating the study intervention characteristics and comparing against the planned groups for each synthesis (item #5)).	Manuscript Pages 5–6
	13b	Describe any methods required to prepare the data for presentation or synthesis, such as handling of missing summary statistics, or data conversions.	Manuscript Pages 5–6
	13c	Describe any methods used to tabulate or visually display results of individual studies and syntheses.	Manuscript Pages 5–6
	13d	Describe any methods used to synthesize results and provide a rationale for the choice(s). If meta-analysis was performed, describe the model(s), method(s) to identify the presence and extent of statistical heterogeneity, and software package(s) used.	Manuscript Pages 5–6
	13e	Describe any methods used to explore possible causes of heterogeneity among study results (e.g. subgroup analysis, meta-regression).	Manuscript Pages 5–6
	13f	Describe any sensitivity analyses conducted to assess robustness of the synthesized results.	Manuscript Pages 5–7
Reporting bias assessment	14	Describe any methods used to assess risk of bias due to missing results in a synthesis (arising from reporting biases).	Manuscript Page 7
Certainty assessment	15	Describe any methods used to assess certainty (or confidence) in the body of evidence for an outcome.	Manuscript Page 7
**RESULTS**			
Study selection	16a	Describe the results of the search and selection process, from the number of records identified in the search to the number of studies included in the review, ideally using a flow diagram.	Manuscript Pages 7–8

	16b	Cite studies that might appear to meet the inclusion criteria, but which were excluded, and explain why they were excluded.	Manuscript Pages 7–8
Study characteristics	17	Cite each included study and present its characteristics.	Manuscript Pages 7–8
Risk of bias in studies	18	Present assessments of risk of bias for each included study.	Supplementary Materials Pages 9-10
Results of individual studies	19	For all outcomes, present, for each study: (a) summary statistics for each group (where appropriate) and (b) an effect estimate and its precision (e.g. confidence/credible interval), ideally using structured tables or plots.	Manuscript Pages 8–9
Results of syntheses	20a	For each synthesis, briefly summarise the characteristics and risk of bias among contributing studies.	Manuscript Pages 8–9
	20b	Present results of all statistical syntheses conducted. If meta-analysis was done, present for each the summary estimate and its precision (e.g. confidence/credible interval) and measures of statistical heterogeneity. If comparing groups, describe the direction of the effect.	Manuscript Pages 8–9
	20c	Present results of all investigations of possible causes of heterogeneity among study results.	Manuscript Pages 9–10
	20d	Present results of all sensitivity analyses conducted to assess the robustness of the synthesized results.	Manuscript Pages 9–10
Reporting biases	21	Present assessments of risk of bias due to missing results (arising from reporting biases) for each synthesis assessed.	Supplementary Materials Pages 10-29
Certainty of evidence	22	Present assessments of certainty (or confidence) in the body of evidence for each outcome assessed.	Supplementary Materials Pages 10-29
**DISCUSSION**			
Discussion	23a	Provide a general interpretation of the results in the context of other evidence.	Manuscript Pages 10–12
	23b	Discuss any limitations of the evidence included in the review.	Manuscript Pages 10–13
	23c	Discuss any limitations of the review processes used.	Manuscript Pages 12-13
	23d	Discuss implications of the results for practice, policy, and future research.	Manuscript Pages 12-13
**OTHER INFORMATION**			

Registration and protocol	24a	Provide registration information for the review, including register name and registration number, or state that the review was not registered.	Manuscript Page 3
	24b	Indicate where the review protocol can be accessed, or state that a protocol was not prepared.	Manuscript Page 3
	24c	Describe and explain any amendments to information provided at registration or in the protocol.	Manuscript Page 3
Support	25	Describe sources of financial or non-financial support for the review, and the role of the funders or sponsors in the review.	Title page 2
Competing interests	26	Declare any competing interests of review authors.	Manuscript Page 13
Availability of data, code and other materials	27	Report which of the following are publicly available and where they can be found: template data collection forms; data extracted from included studies; data used for all analyses; analytic code; any other materials used in the review.	Manuscript Page 13

Table S2

Electronic database search (most recent search date: 12 November 2024)

**Information source**	PubMed
**Search strategy**	("cognitive training"[tiab] OR "cognitive rehabilitation"[tiab] OR "cognitive therapy"[tiab] OR "neuropsychological therapy"[tiab] OR "cognitive intervention"[tiab] OR "executive function training"[tiab] OR "working memory training"[tiab] OR "memory training"[tiab] OR "attention training"[tiab] OR "processing speed training"[tiab] OR "brain training"[tiab] OR "computer-based cognitive training"[tiab] OR "virtual reality cognitive training"[tiab] OR "multitasking training"[tiab] OR "dual task training"[tiab] OR "cognitive flexibility training"[tiab] OR "inhibitory control training"[tiab] OR "switching training"[tiab] OR "n-back training"[tiab] OR "updating training"[tiab] OR "shifting training"[tiab] OR "flexibility training"[tiab] OR "multi-domain training"[tiab] OR "cognitive adaptation training"[tiab] OR "brain fitness"[tiab] OR "brain games"[tiab] OR "computerized cognitive training"[tiab]) AND ("fMRI"[tiab] OR "functional magnetic resonance imaging"[tiab] OR "PET"[tiab] OR "positron emission tomography"[tiab] OR "SPECT"[tiab] OR "single photon emission computed tomography"[tiab] OR "functional imaging"[tiab] OR "neuroimaging"[tiab] OR "neuroplasticity"[tiab] OR "neural activation"[tiab] OR "brain function"[tiab])
**No. of records**	700

**Information source**	MEDLINE
**Search strategy**	("cognitive training".ab,ti OR "cognitive rehabilitation".ab,ti OR "cognitive therapy".ab,ti OR "neuropsychological therapy".ab,ti OR "cognitive intervention".ab,ti OR "executive function training".ab,ti OR "working memory training".ab,ti OR "memory training".ab,ti OR "attention training".ab,ti OR "processing speed training".ab,ti OR "brain training".ab,ti OR "computer-based cognitive training".ab,ti OR "virtual reality cognitive training".ab,ti OR "multitasking training".ab,ti OR "dual task training".ab,ti OR "cognitive flexibility training".ab,ti OR "inhibitory control training".ab,ti OR "switching training".ab,ti OR "n-back training".ab,ti OR "updating training".ab,ti OR "shifting training".ab,ti OR "flexibility training".ab,ti OR "multi-domain training".ab,ti OR "cognitive adaptation training".ab,ti OR "brain fitness".ab,ti OR "brain games".ab,ti OR "computerized cognitive training".ab,ti) AND ("fMRI".ab,ti OR "functional magnetic resonance imaging".ab,ti OR "PET".ab,ti OR "positron emission tomography".ab,ti OR "SPECT".ab,ti OR "single photon emission computed tomography".ab,ti OR "functional imaging".ab,ti OR "neuroimaging".ab,ti OR "neuroplasticity".ab,ti OR "neural

	activation".ab,ti OR "brain function".ab,ti)
**No. of records**	165

**Information source**	PsycINFO
**Search strategy**	("cognitive training".ti,ab OR "cognitive rehabilitation".ti,ab OR "cognitive therapy".ti,ab OR "neuropsychological therapy".ti,ab OR "cognitive intervention".ti,ab OR "executive function training".ti,ab OR "working memory training".ti,ab OR "memory training".ti,ab OR "attention training".ti,ab OR "processing speed training".ti,ab OR "brain training".ti,ab OR "computer-based cognitive training".ti,ab OR "virtual reality cognitive training".ti,ab OR "multitasking training".ti,ab OR "dual task training".ti,ab OR "cognitive flexibility training".ti,ab OR "inhibitory control training".ti,ab OR "switching training".ti,ab OR "n-back training".ti,ab OR "updating training".ti,ab OR "shifting training".ti,ab OR "flexibility training".ti,ab OR "multi-domain training".ti,ab OR "cognitive adaptation training".ti,ab OR "brain fitness".ti,ab OR "brain games".ti,ab OR "computerized cognitive training".ti,ab) AND ("fMRI".ti,ab OR "functional magnetic resonance imaging".ti,ab OR "PET".ti,ab OR "positron emission tomography".ti,ab OR "SPECT".ti,ab OR "single photon emission computed tomography".ti,ab OR "functional imaging".ti,ab OR "neuroimaging".ti,ab OR "neuroplasticity".ti,ab OR "neural activation".ti,ab OR "brain function".ti,ab)
**No. of records**	1,025

**Information source**	Embase
**Search strategy**	("cognitive training".ti,ab OR "cognitive rehabilitation".ti,ab OR "cognitive therapy".ti,ab OR "neuropsychological therapy".ti,ab OR "cognitive intervention".ti,ab OR "executive function training".ti,ab OR "working memory training".ti,ab OR "memory training".ti,ab OR "attention training".ti,ab OR "processing speed training".ti,ab OR "brain training".ti,ab OR "computer-based cognitive training".ti,ab OR "virtual reality cognitive training".ti,ab OR "multitasking training".ti,ab OR "dual task training".ti,ab OR "cognitive flexibility training".ti,ab OR "inhibitory control training".ti,ab OR "switching training".ti,ab OR "n-back training".ti,ab OR "updating training".ti,ab OR "shifting training".ti,ab OR "flexibility training".ti,ab OR "multi-domain training".ti,ab OR "cognitive adaptation training".ti,ab OR "brain fitness".ti,ab OR "brain games".ti,ab OR "computerized cognitive training".ti,ab) AND ("fMRI".ti,ab OR "functional magnetic resonance imaging".ti,ab OR "PET".ti,ab OR "positron emission tomography".ti,ab OR "SPECT".ti,ab OR "single photon emission

	computed tomography".ti,ab OR "functional imaging".ti,ab OR "neuroimaging".ti,ab OR "neuroplasticity".ti,ab OR "neural activation".ti,ab OR "brain function".ti,ab)
**No. of records**	1,334

**Information source**	Web of science
**Search strategy**	("cognitive training" OR "cognitive rehabilitation" OR "cognitive therapy" OR "neuropsychological therapy" OR "cognitive intervention" OR "executive function training" OR "working memory training" OR "memory training" OR "attention training" OR "processing speed training" OR "brain training" OR "computer-based cognitive training" OR "virtual reality cognitive training" OR "multitasking training" OR "dual task training" OR "cognitive flexibility training" OR "inhibitory control training" OR "switching training" OR "n-back training" OR "updating training" OR "shifting training" OR "flexibility training" OR "multi-domain training" OR "cognitive adaptation training" OR "brain fitness" OR "brain games" OR "computerized cognitive training") AND ("fMRI" OR "functional magnetic resonance imaging" OR "PET" OR "positron emission tomography" OR "SPECT" OR "single photon emission computed tomography" OR "functional imaging" OR "neuroimaging" OR "neuroplasticity" OR "neural activation" OR "brain function")
**No. of records**	1,435

Table S3

Quality assessment checklist

Subjects

1. The sample size was appropriate

2. Subjects were evaluated prospectively, demographic data were reported (age (mean and SD/range), sex, and handedness), and psychiatric and medical illnesses were excluded

3. If any subject was scanned but then rejected from the analysis, withdrawals from the study were explained

Methods for tasks

4. All participants went through a training session outside the scanner

5. The experimental design was meticulously detailed to enable replication, including the number of blocks or trials per participant, the duration of each trial, the inter-stimulus interval, and whether the design was block-based or event-related.

6. The stimuli and the number of repetitions were sufficient and clearly described

7. If applicable, the baseline condition was defined as almost the same as the task condition.

Methods for image acquisition and statistical analysis

8. MRI slice thickness $\leq$ 3 mm

9. 3T MRI was used

10. The imaging technique used for data acquisition was clearly described so that it could be reproduced (e.g., MRI system used, field strength, pulse sequence type, number of volumes per session, field of view, matrix size, slice thickness, interslice skip, acquisition orientation, TE/TR/flip angle)

11. Preprocessing operations were clearly described and detailed so that they could be reproduced (e.g., software used, order of preprocessing operations, slice-timing, motion correction, coregistration and normalization (linear/affine or nonlinear), smoothing)

12. Adjustments were made for multiple statistical comparisons

13. Appropriate design and/or analytical methods to control confounding

14. Appropriate use of statistics for primary analysis effect (excluding control of confounders)

Results, conclusions, and conflicts of interest

15. Statistical parameters for significant and important nonsignificant differences were provided

16. Conclusions were consistent with the results obtained and the limitations were discussed	
17. Declarations of conflicts of interest or identification of funding sources	

Note. Score 0/0.5/1 for each item (0.5 points were given for criteria partially met).

Table S4

Participant, intervention, result and quality of included studies

No.	Study	Population (mean age)	Intervention group (n)	Intervention details	Control group (n)	In-scan tasks	Qualit y
1	Belleville et al., 2011	Patients with MCI (70.13)	Memory training (30)	Session duration: 120 minutes<br>Program length: 6 weeks<br>Training frequency: 1 time per week<br>Training domain: Single-domain<br>Training paradigm: Strategy-based<br>Delivery mode: Group-based<br>Difficulty regulation: Adaptive	None	Memory encoding and retrieval task	16.5
2	Belleville et al., 2023	Patients with MCI (67)	Multi-domain training (29)	Session duration: 60 minutes<br>Program length: 2 weeks<br>Training frequency: 3 times per week<br>Training domain: Multi-domain<br>Training paradigm: Strategy-based<br>Delivery mode: Group-based<br>Difficulty regulation: Adaptive	None	Word list encoding and recognition task	15
3	Bonzano et al., 2020	Patients with multiple sclerosis (45.3)	Working memory training (30)	Session duration: 30 minutes<br>Program length: 8 weeks<br>Training frequency: 5 times per week<br>Training domain: Single-domain<br>Training paradigm: Process-based<br>Delivery mode: Individual-based	None	Paced visual serial addition task	16

				Difficulty regulation: Adaptive			
4	Campbell et al., 2016	Patients with multiple sclerosis (47.37)	Multi-domain training (19)	Session duration: 45 minutes<br>Program length: 6 weeks<br>Training frequency: 3 times per week<br>Training domain: Multi-domain<br>Training paradigm: Process-based<br>Delivery mode: Individual-based<br>Difficulty regulation: Adaptive	Watched nature documentaries (19)	N-back task	15.5
5	Cerasa et al., 2013	Patients with multiple sclerosis (32.7)	Attention training (12)	Session duration: 60 minutes<br>Program length: 6 weeks<br>Training frequency: 2 times per week<br>Training domain: Single-domain<br>Training paradigm: Process-based<br>Delivery mode: Individual-based<br>Difficulty regulation: Adaptive	Education program (11)	Paced visual serial addition task	17
6	Chen et al., 2020	Patients with MCI (74.6)	Processing speech training (46)	Session duration: 60 minutes<br>Program length: 6 weeks<br>Training frequency: 4 times per week<br>Training domain: Single-domain<br>Training paradigm: Process-based<br>Delivery mode: Individual-based<br>Difficulty regulation: Adaptive	Mental leisure activities (38)	Visual search task	17

7	Chiaravalloti et al., 2015	Patients with TBI (44)	Memory training (9)	Session duration: 52.5 minutes<br>Program length: 5 weeks<br>Training frequency: 2 times per week<br>Training domain: Single-domain<br>Training paradigm: Strategy-based<br>Delivery mode: Individual-based<br>Difficulty regulation: Non-adaptive	Sham training (9)	Recall task	16
8	Ekman et al., 2018	Patients with stroke (72.7)	Visual scanning training (12)	Session duration: 60 minutes<br>Program length: 5 weeks<br>Training frequency: 3 times per week<br>Training domain: Single-domain<br>Training paradigm: Process-based<br>Delivery mode: Individual-based<br>Difficulty regulation: Adaptive	None	Posner cuing task	17
9	Garriga et al., 2010	Patients with multiple sclerosis (50.73)	Multi-domain training (15)	Session duration: 60 minutes<br>Program length: 5 weeks<br>Training frequency: 4 times per week<br>Training domain: Multi-domain<br>Training paradigm: Process-based<br>Delivery mode: NA	No training (5)	Paced auditory serial addition task	16.5

				Difficulty regulation: Adaptive			
10	Hampstead et al., 2011	Patients with MCI (66.04)	Working memory training (15)	Session duration: 30 minutes<br>Program length: 2 weeks<br>Training frequency: 5 times per week<br>Training domain: Single-domain<br>Training paradigm: Strategy-based<br>Delivery mode: Individual-based<br>Difficulty regulation: Non-adaptive	No training (14)	T1: N-back task<br>T2: Sternberg delayed match-to-sample task	15
11	Hampstead et al., 2020	Patients with MCI (72.1)	G1: Mnemonic strategy-based training (9)<br>G2: Mnemonic process -based training (9)	Session duration: 30 minutes<br>Program length: 2 weeks<br>Training frequency: 5 times per week<br>Training domain: Single-domain<br>Training paradigm:Strategy-based in G1<br>Process-based in G2<br>Delivery mode: Individual-based<br>Difficulty regulation: Non-adaptive	None	Object–location association encoding task	16.5

12	Huntley et al., 2017	Patients with AD (79.77)	Working memory training (15)	Session duration: 30 minutes<br>Program length: 8 weeks<br>Training frequency: 2–3 times per week<br>Training domain: Single-domain<br>Training paradigm: Strategy-based<br>Delivery mode: Individual-based<br>Difficulty regulation: Adaptive	Low-difficulty training (15)	Verbal working memory task	17
13	Kim et al., 2009	Patients with TBI (30.1)	Attention training (10)	Session duration: 30 minutes<br>Program length: 4 weeks<br>Training frequency: 3 times per week<br>Training domain: Single-domain<br>Training paradigm: Process-based<br>Delivery mode: Individual-based<br>Difficulty regulation: Adaptive	None	Endogenous visuospatial attention task	17
14	Miotto et al., 2013	Patients with prefrontal cortex lesions (42.65)	Strategic semantic organizational training (21)	Session duration: 30 minutes<br>Program length: 1 week<br>Training frequency: 1 times per week<br>Training domain: Single-domain<br>Training paradigm: Strategy-based<br>Delivery mode: Individual-based<br>Difficulty regulation: Adaptive	None	Verbal memory encoding task	17

15	Miotto et al., 2020	Patients with stroke patients (38.35)	Semantic memory training (12)	Session duration: 90 minutes<br>Program length: 2 weeks<br>Training frequency: 1–2 times per week<br>Training domain: Single-domain<br>Training paradigm: Strategy-based<br>Delivery mode: Individual-based<br>Difficulty regulation: Non-adaptive	Education program (13)	Word-List learning task	17
16	Paasschen et al., 2013	Patients with AD (74.71)	Multi-domain training (7)	Session duration: 60 minutes<br>Program length: 8 weeks<br>Training frequency: 1 time per week<br>Training domain: Single-domain<br>Training paradigm: Strategy-based<br>Delivery mode: Individual-based<br>Difficulty regulation: Adaptive	No training (12)	Associative memory task	14
17	Paradela et al., 2023	Patients with MCI (58.22)	Working memory training (17)	Session duration: 35 minutes<br>Program length: 10 weeks<br>Training frequency: 3 times per week<br>Training domain: Single-domain<br>Training paradigm: Process-based<br>Delivery mode: Individual-based<br>Difficulty regulation: Adaptive	Low-difficulty training (15)	Working memory task with low and high load	17

18	Rocca et al., 2024	Patients with progressive multiple sclerosis (52.5)	Multi-domain training (27)	Session duration: 45–60 minutes<br>Program length: 12 weeks<br>Training frequency: 3 times per week<br>Training domain: Single-domain<br>Training paradigm: Process-based<br>Delivery mode: Group-based<br>Difficulty regulation: Adaptive	None	Go/nogo task	16.5
19	Rosen et al., 2011	Patients with MCI(74.34)	Processing speed training (6)	Session duration: 20–100 minutes<br>Program length: 8 weeks<br>Training frequency: 5 times per week<br>Training domain: Single-domain<br>Training paradigm: Process-based<br>Delivery mode: Individual-based<br>Difficulty regulation: Adaptive	Mental leisure activities (6)	Auditory memory task	
20	Simon et al., 2018	Patients with MCI (72.15)	Memory training (15)	Session duration: 45 minutes<br>Program length: 2 weeks<br>Training frequency: 4 times per week<br>Training domain: Single-domain<br>Training paradigm: Process-based<br>Delivery mode: Strategy-based<br>Difficulty regulation: Non-daptive	Education program (15)	Face-name recognition task	17

21	Simon et al., 2020	Patients with MCI (23.9)	Memory training (15)	Session duration: 60 minutes<br>Program length: 2 weeks<br>Training frequency: 2 times per week<br>Training domain: Single-domain<br>Training paradigm: Strategy-based<br>Delivery mode: Individual-based<br>Difficulty regulation: Non-daptive	None	Face-name recognition task	17

Note. n = number of participants included; G = group; T = task; TBI = traumatic brain injury; MCI = Mild Cognitive Impairment; AD = Alzheimer Disease.

Table S5

Results of the jackknife analysis in all included studies

**Jackknife sensitivity analysis , discarded study**	**Precuneus**	**L. PreCG**
Belleville et al., 2011	Yes	Yes
Belleville et al., 2023	Yes	Yes
Bonzano et al., 2020	Yes	Yes
Campbell et al., 2016	Yes	Yes
Cerasa et al., 2013	Yes	Yes
Chen et al., 2020	Yes	Yes
Chiaravalloti et al., 2015	Yes	Yes
Ekman et al., 2018	Yes	Yes
Garriga et al., 2010	Yes	Yes
Hampstead et al., 2011	Yes	Yes
Hampstead et al., 2020	Yes	Yes
Huntley et al., 2017	Yes	Yes
Kim et al., 2009	Yes	Yes
Miotto et al., 2013	Yes	Yes
Miotto et al., 2020	Yes	Yes
Paasschen et al., 2013	Yes	Yes
Paradela et al., 2023	Yes	Yes
Rocca et al., 2024	Yes	Yes
Rosen et al., 2011	Yes	Yes
Simon et al., 2018	Yes	Yes
Simon et al., 2020	Yes	Yes

Note. Jackknife sensitivity analysis was conducted by iteratively excluding one study at a time to assess the robustness and reliability of the main findings. The consistent “Yes” results across all included studies indicate that the significant clusters in the precuneus and L. PreCG remained stable and were not driven by any single study. L. PreCG = left precentral gyrus.

Table S6

Sensitivity results of multivariate meta-analysis across assumed within-study correlations

r	Hedges' g	SE	CI_lower	CI_upper	p_value	t_value
0.3	.4641	.1214	.2183	.7098	.0005	3.8231
0.7	.4369	.1289	.1760	.6977	.0016	3.3903
1	.4264	.1473	.1281	.7246	.0063	2.8938

Note. Sensitivity analyses of the multivariate meta-analysis were performed under different assumptions of within-study correlations (r = 0.3, 0.7, and 1). The results consistently demonstrated significant effect sizes, with only minimal variation in Hedges' g, standard errors, and confidence intervals across correlation levels. This indicates that the overall findings are robust and not substantially influenced by the assumed within-study correlation.

Figure S1

Funnel plot for bilateral precuneus

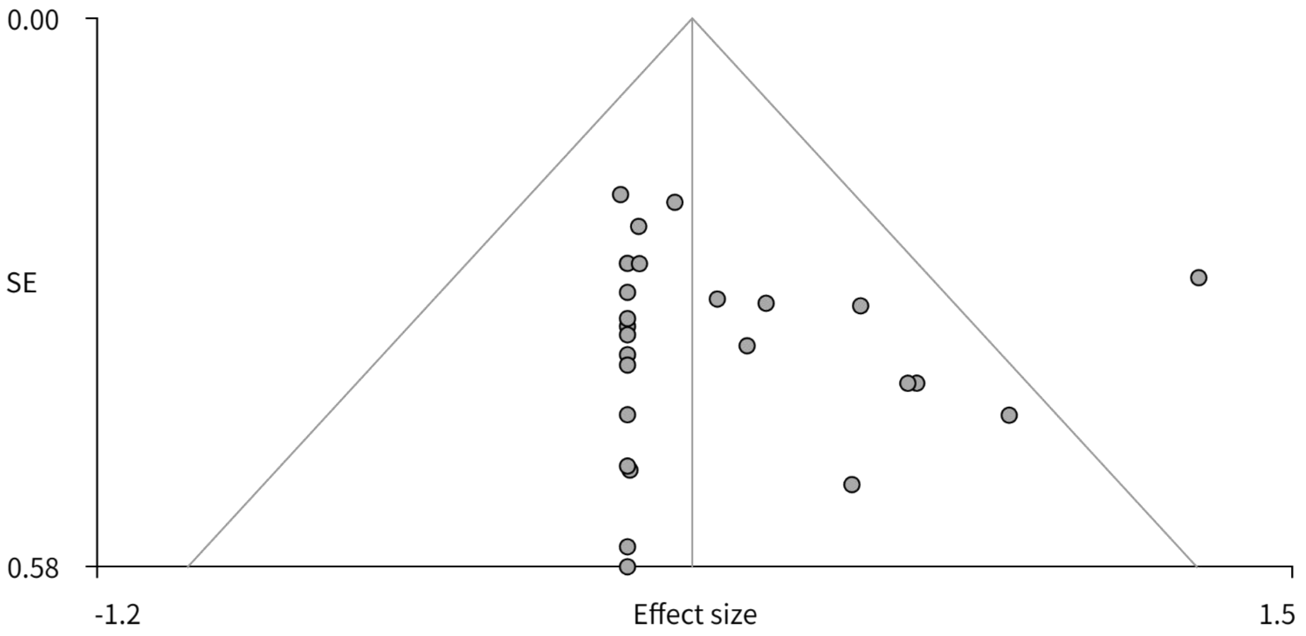

Note. Funnel plot of study-level effect sizes versus standard errors. Egger's test: $\beta = 0.28$, $t = 0.46$, $p = 0.65$.

Figure S2

Funnel plot for L. PreCG

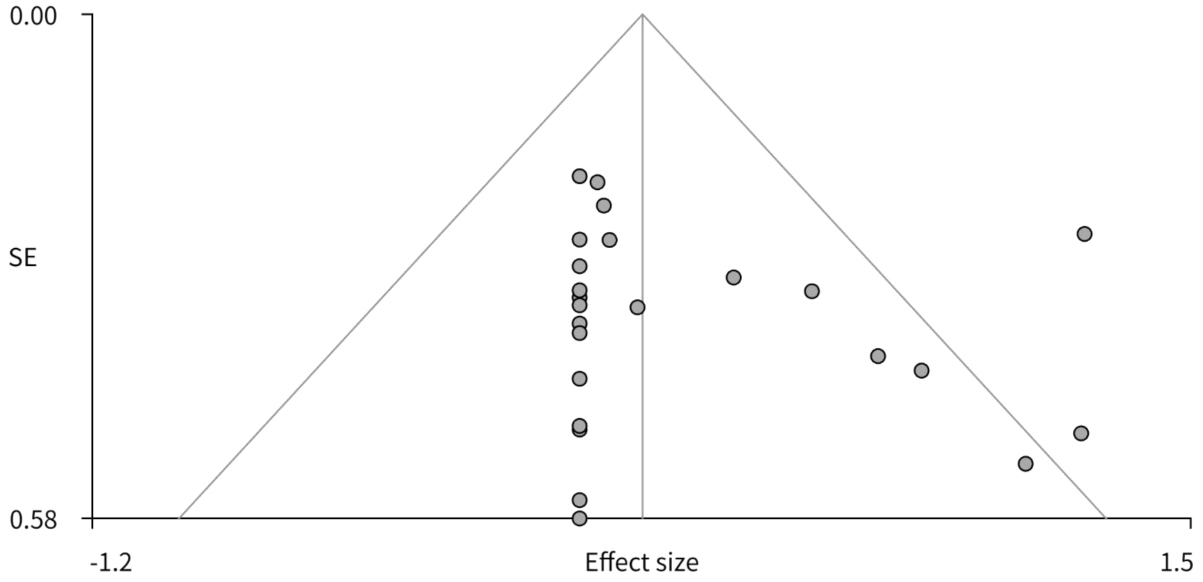

Note. Funnel plot of study-level effect sizes versus standard errors. Egger's test: $\beta = 0.56$, $t = 0.84$, $p = 0.405$. L. PreCG = left precentral gyrus.
